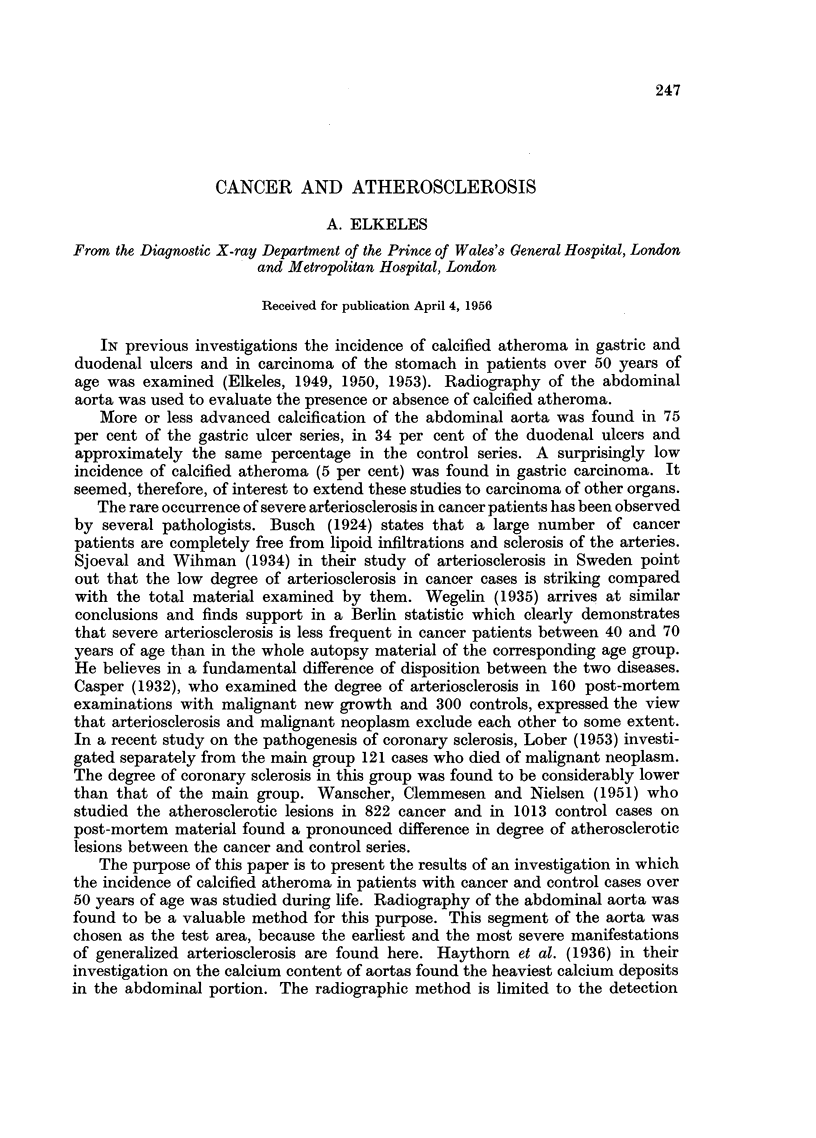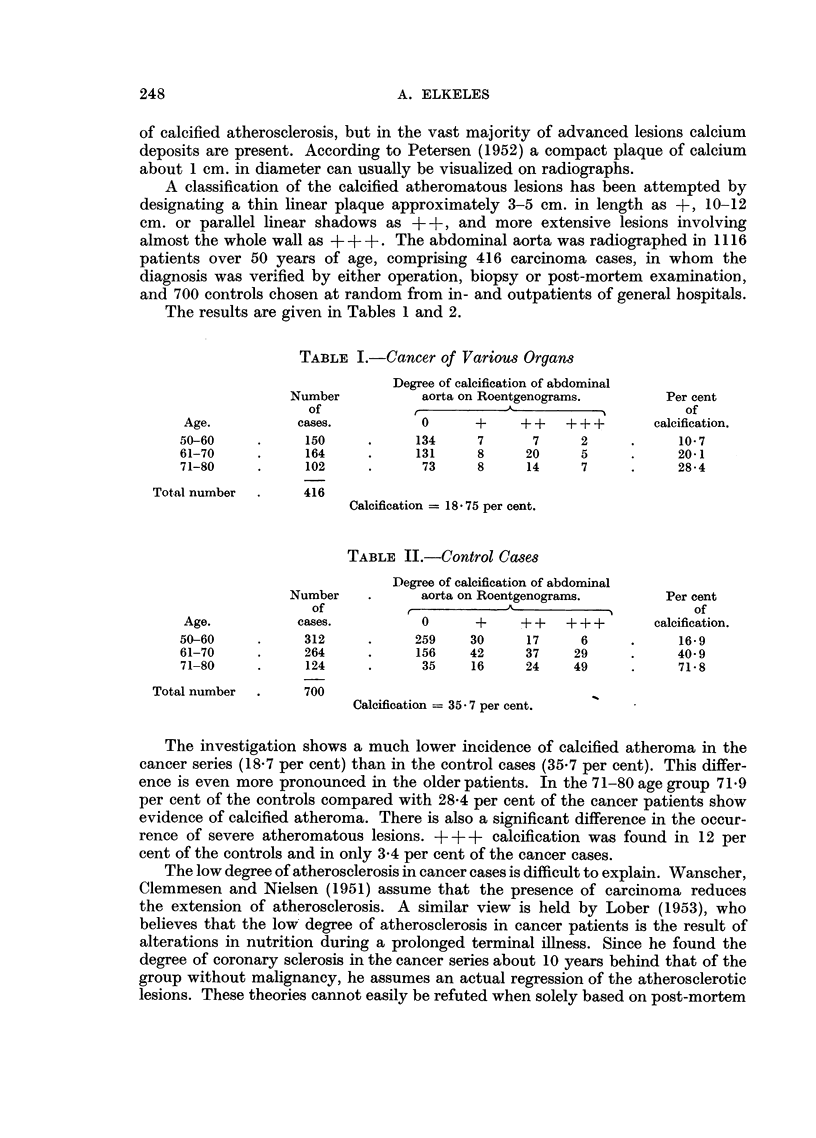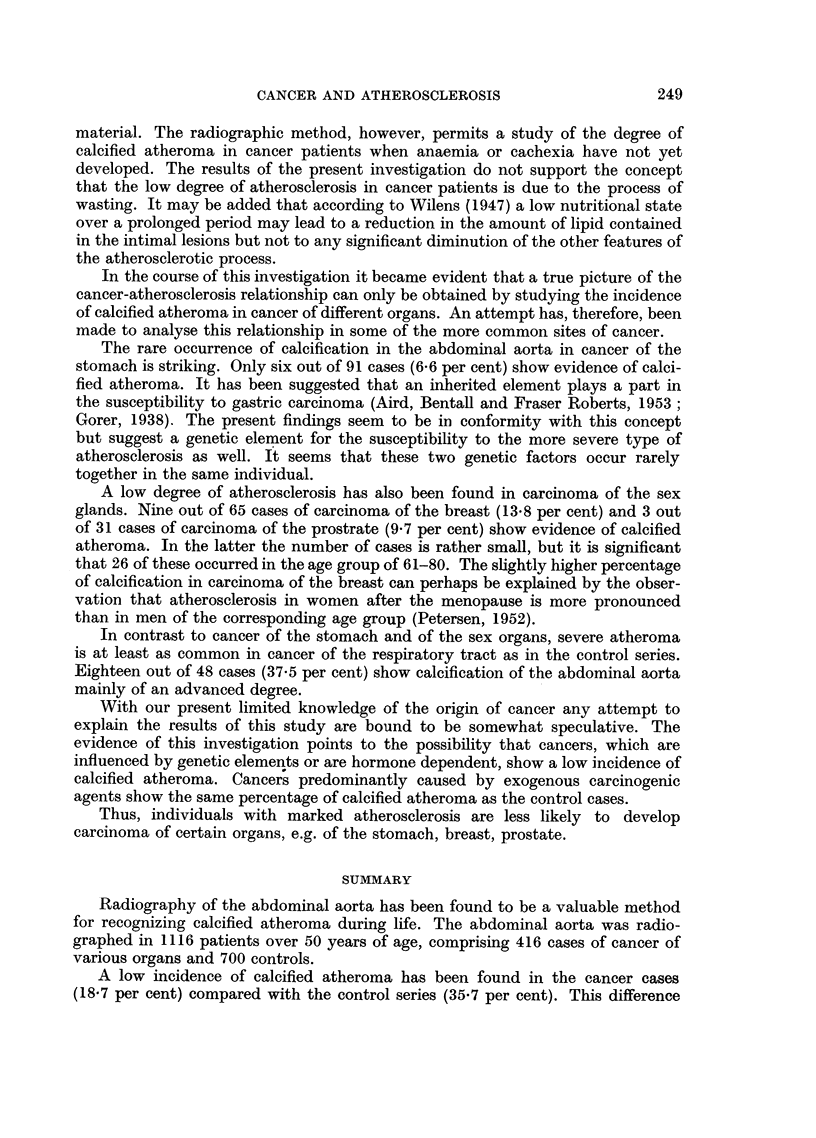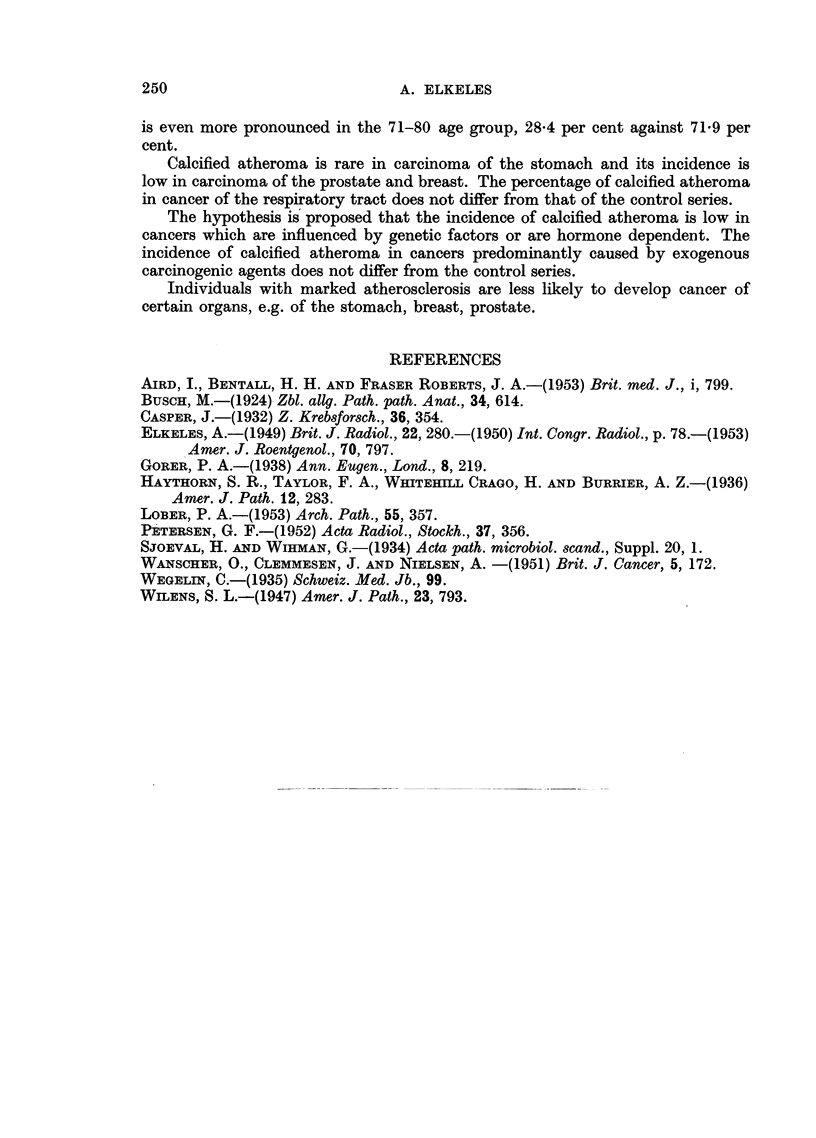# Cancer and Atherosclerosis

**DOI:** 10.1038/bjc.1956.28

**Published:** 1956-06

**Authors:** A. Elkeles


### CANCER AND ATHEROSCLEROSIS

A. ELKELES

*From the Diagnostic X-ray Department of the Prince of Wales's General Hospital, London and Metropolitan Hospital, London*

Received for publication April 4, 1956

In previous investigations the incidence of calcified atheroma in gastric and duodenal ulcers and in carcinoma of the stomach in patients over 50 years of age was examined (Elkeles, 1949, 1950, 1953). Radiography of the abdominal aorta was used to evaluate the presence or absence of calcified atheroma.

More or less advanced calcification of the abdominal aorta was found in 75 per cent of the gastric ulcer series, in 34 per cent of the duodenal ulcers and approximately the same percentage in the control series. A surprisingly low incidence of calcified atheroma (5 per cent) was found in gastric carcinoma. It seemed, therefore, of interest to extend these studies to carcinoma of other organs.

The rare occurrence of severe arteriosclerosis in cancer patients has been observed by several pathologists. Busch (1924) states that a large number of cancer patients are completely free from lipoid infiltrations and sclerosis of the arteries. Sjoeval and Wihman (1934) in their study of arteriosclerosis in Sweden point out that the low degree of arteriosclerosis in cancer cases is striking compared with the total material examined by them. Wegelin (1935) arrives at similar conclusions and finds support in a Berlin statistic which clearly demonstrates that severe arteriosclerosis is less frequent in cancer patients between 40 and 70 years of age than in the whole autopsy material of the corresponding age group. He believes in a fundamental difference of disposition between the two diseases. Casper (1932), who examined the degree of arteriosclerosis in 160 post-mortem examinations with malignant new growth and 300 controls, expressed the view that arteriosclerosis and malignant neoplasm exclude each other to some extent. In a recent study on the pathogenesis of coronary sclerosis, Lober (1953) investigated separately from the main group 121 cases who died of malignant neoplasm. The degree of coronary sclerosis in this group was found to be considerably lower than that of the main group. Wanscher, Clemmesen and Nielsen (1951) who studied the atherosclerotic lesions in 822 cancer and in 1013 control cases on post-mortem material found a pronounced difference in degree of atherosclerotic lesions between the cancer and control series.

The purpose of this paper is to present the results of an investigation in which the incidence of calcified atheroma in patients with cancer and control cases over 50 years of age was studied during life. Radiography of the abdominal aorta was found to be a valuable method for this purpose. This segment of the aorta was chosen as the test area, because the earliest and the most severe manifestations of generalized arteriosclerosis are found here. Haythorn *et al.* (1936) in their investigation on the calcium content of aortas found the heaviest calcium deposits in the abdominal portion. The radiographic method is limited to the detection of calcified atherosclerosis, but in the vast majority of advanced lesions calcium deposits are present. According to Petersen (1952) a compact plaque of calcium about 1 cm. in diameter can usually be visualized on radiographs.

A classification of the calcified atheromatous lesions has been attempted by designating a thin linear plaque approximately 3–5 cm. in length as +, 10–12 cm. or parallel linear shadows as ++, and more extensive lesions involving almost the whole wall as +++. The abdominal aorta was radiographed in 1116 patients over 50 years of age, comprising 416 carcinoma cases, in whom the diagnosis was verified by either operation, biopsy or post-mortem examination, and 700 controls chosen at random from in- and outpatients of general hospitals. The results are given in Tables 1 and 2.

TABLE I.—*Cancer of Various Organs*

| Age. | Number of cases. | Degree of calcification of abdominal aorta on Roentgenograms. 0 | + | ++ | +++ | Per cent of calcification. |
|---|---|---|---|---|---|---|
| 50–60 | 150 | 134 | 7 | 7 | 2 | 10·7 |
| 61–70 | 164 | 131 | 8 | 20 | 5 | 20·1 |
| 71–80 | 102 | 73 | 8 | 14 | 7 | 28·4 |
| Total number | 416 | | | | | |

Calcification = 18·75 per cent.

TABLE II.—*Control Cases*

| Age. | Number of cases. | Degree of calcification of abdominal aorta on Roentgenograms. 0 | + | ++ | +++ | Per cent of calcification. |
|---|---|---|---|---|---|---|
| 50–60 | 312 | 259 | 30 | 17 | 6 | 16·9 |
| 61–70 | 264 | 156 | 42 | 37 | 29 | 40·9 |
| 71–80 | 124 | 35 | 16 | 24 | 49 | 71·8 |
| Total number | 700 | | | | | |

Calcification = 35·7 per cent.

The investigation shows a much lower incidence of calcified atheroma in the cancer series (18·7 per cent) than in the control cases (35·7 per cent). This difference is even more pronounced in the older patients. In the 71–80 age group 71·9 per cent of the controls compared with 28·4 per cent of the cancer patients show evidence of calcified atheroma. There is also a significant difference in the occurrence of severe atheromatous lesions. +++ calcification was found in 12 per cent of the controls and in only 3·4 per cent of the cancer cases.

The low degree of atherosclerosis in cancer cases is difficult to explain. Wanscher, Clemmesen and Nielsen (1951) assume that the presence of carcinoma reduces the extension of atherosclerosis. A similar view is held by Lober (1953), who believes that the low degree of atherosclerosis in cancer patients is the result of alterations in nutrition during a prolonged terminal illness. Since he found the degree of coronary sclerosis in the cancer series about 10 years behind that of the group without malignancy, he assumes an actual regression of the atherosclerotic lesions. These theories cannot easily be refuted when solely based on post-mortem material. The radiographic method, however, permits a study of the degree of calcified atheroma in cancer patients when anaemia or cachexia have not yet developed. The results of the present investigation do not support the concept that the low degree of atherosclerosis in cancer patients is due to the process of wasting. It may be added that according to Wilens (1947) a low nutritional state over a prolonged period may lead to a reduction in the amount of lipid contained in the intimal lesions but not to any significant diminution of the other features of the atherosclerotic process.

In the course of this investigation it became evident that a true picture of the cancer-atherosclerosis relationship can only be obtained by studying the incidence of calcified atheroma in cancer of different organs. An attempt has, therefore, been made to analyse this relationship in some of the more common sites of cancer.

The rare occurrence of calcification in the abdominal aorta in cancer of the stomach is striking. Only six out of 91 cases (6·6 per cent) show evidence of calcified atheroma. It has been suggested that an inherited element plays a part in the susceptibility to gastric carcinoma (Aird, Bentall and Fraser Roberts, 1953 ; Gorer, 1938). The present findings seem to be in conformity with this concept but suggest a genetic element for the susceptibility to the more severe type of atherosclerosis as well. It seems that these two genetic factors occur rarely together in the same individual.

A low degree of atherosclerosis has also been found in carcinoma of the sex glands. Nine out of 65 cases of carcinoma of the breast (13·8 per cent) and 3 out of 31 cases of carcinoma of the prostrate (9·7 per cent) show evidence of calcified atheroma. In the latter the number of cases is rather small, but it is significant that 26 of these occurred in the age group of 61–80. The slightly higher percentage of calcification in carcinoma of the breast can perhaps be explained by the observation that atherosclerosis in women after the menopause is more pronounced than in men of the corresponding age group (Petersen, 1952).

In contrast to cancer of the stomach and of the sex organs, severe atheroma is at least as common in cancer of the respiratory tract as in the control series. Eighteen out of 48 cases (37·5 per cent) show calcification of the abdominal aorta mainly of an advanced degree.

With our present limited knowledge of the origin of cancer any attempt to explain the results of this study are bound to be somewhat speculative. The evidence of this investigation points to the possibility that cancers, which are influenced by genetic elements or are hormone dependent, show a low incidence of calcified atheroma. Cancers predominantly caused by exogenous carcinogenic agents show the same percentage of calcified atheroma as the control cases.

Thus, individuals with marked atherosclerosis are less likely to develop carcinoma of certain organs, e.g. of the stomach, breast, prostate.

### SUMMARY

Radiography of the abdominal aorta has been found to be a valuable method for recognizing calcified atheroma during life. The abdominal aorta was radiographed in 1116 patients over 50 years of age, comprising 416 cases of cancer of various organs and 700 controls.

A low incidence of calcified atheroma has been found in the cancer cases (18·7 per cent) compared with the control series (35·7 per cent). This difference is even more pronounced in the 71–80 age group, 28·4 per cent against 71·9 per cent.

Calcified atheroma is rare in carcinoma of the stomach and its incidence is low in carcinoma of the prostate and breast. The percentage of calcified atheroma in cancer of the respiratory tract does not differ from that of the control series.

The hypothesis is proposed that the incidence of calcified atheroma is low in cancers which are influenced by genetic factors or are hormone dependent. The incidence of calcified atheroma in cancers predominantly caused by exogenous carcinogenic agents does not differ from the control series.

Individuals with marked atherosclerosis are less likely to develop cancer of certain organs, e.g. of the stomach, breast, prostate.

### REFERENCES

Aird, I., Bentall, H. H. and Fraser Roberts, J. A.—(1953) *Brit. med. J.*, i, 799.
Busch, M.—(1924) *Zbl. allg. Path. path. Anat.*, **34**, 614.
Casper, J.—(1932) *Z. Krebsforsch.*, **36**, 354.
Elkeles, A.—(1949) *Brit. J. Radiol.*, **22**, 280.—(1950) *Int. Congr. Radiol.*, p. 78.—(1953) *Amer. J. Roentgenol.*, **70**, 797.
Gorer, P. A.—(1938) *Ann. Eugen., Lond.*, **8**, 219.
Haythorn, S. R., Taylor, F. A., Whitehill Crago, H. and Burrier, A. Z.—(1936) *Amer. J. Path.* **12**, 283.
Lober, P. A.—(1953) *Arch. Path.*, **55**, 357.
Petersen, G. F.—(1952) *Acta Radiol., Stockh.*, **37**, 356.
Sjoeval, H. and Wihman, G.—(1934) *Acta path. microbiol. scand.*, Suppl. 20, 1.
Wanscher, O., Clemmesen, J. and Nielsen, A. —(1951) *Brit. J. Cancer*, **5**, 172.
Wegelin, C.—(1935) *Schweiz. Med. Jb.*, **99**.
Wilens, S. L.—(1947) *Amer. J. Path.*, **23**, 793.